# Allelic Analysis of the *Gli-B1* Locus in Hexaploid Wheat Using Reverse-Phase–Ultra-Performance Liquid Chromatography

**DOI:** 10.3390/molecules30030609

**Published:** 2025-01-30

**Authors:** Jong-Yeol Lee, Yu-Jeong Yang, Jinpyo So, Sewon Kim, Kyoungwon Cho

**Affiliations:** 1 National Institute of Agricultural Sciences, Rural Development Administration, Jeonju 54874, Republic of Korea; jy0820@korea.kr (J.-Y.L.); yujinge@korea.kr (Y.-J.Y.); 2 Department of Integrative Food, Bioscience and Biotechnology, College of Agriculture and Life Sciences, Chonnam National University, Gwangju 61166, Republic of Korea; thwlsvy123@gmail.com

**Keywords:** omega-5 gliadin, reverse-phase–ultra-performance liquid chromatography (RP-UPLC), *Gli-B1* locus

## Abstract

Wheat (*Triticum aestivum* L.) omega-5 gliadin, a major allergen responsible for wheat-dependent exercise-induced anaphylaxis in humans, is encoded by genes located at the *Gli-B1* locus on chromosome 1B, which exhibits genetic polymorphism. *Gli-B1* alleles have generally been identified based on the electrophoretic mobilities of the encoded gamma-, omega-1,2, and omega-5 gliadins in acid polyacrylamide gel electrophoresis. However, the similar mobilities of omega-5 gliadin variants make it difficult to distinguish them among different wheat varieties. In this study, we optimized reverse-phase–ultra-performance liquid chromatography (RP-UPLC) conditions to separate omega-5 gliadins in the reference wheat cultivar Chinese Spring and its nullisomic–tetrasomic lines for chromosome 1B. Five chromatographic peaks corresponded to omega-5 gliadin, and the average relative standard deviation to each peak retention time ranged from 0.31% to 0.93%, indicating that the method is accurate and reproducible for fractionating omega-5 gliadins in gliadin extracts from wheat flour. Using the optimized RP-UPLC method, we analyzed omega-5 gliadins in 24 wheat varieties with the *Gli-B1f* allele. The result showed that the wheat varieties were sorted into eight groups according to the composition of omega-5 gliadin, indicating that the classification of *Gli-B1* alleles based on A-PAGE could not explain the composition of omega-5 gliadin in wheat. We reclassified 73 wheat varieties containing 16 unique *Gli-B1* alleles into 31 groups based on the chromatographic patterns of their omega-5 gliadins. Our results provide information on the specific *Gli-B1* alleles of wheat varieties belonging to each group and demonstrate the potential for RP-UPLC to facilitate genetic studies of wheat varieties.

## 1. Introduction

Wheat (*Triticum aestivum* L.) is an important staple grain crop consumed by humans worldwide and is a major source of protein in the daily diet. Wheat flour is used in bread, noodles, cakes, and snacks, and the end-use quality is mainly determined by gluten proteins, the storage proteins of the grain endosperm. Glutens include polymeric glutenins and monomeric gliadins and play a crucial role in providing viscoelasticity and extensibility to wheat dough [1]. Glutenins are composed of high-molecular-weight glutenin subunits (HMW-GSs) and low-molecular-weight glutenin subunits (LMW-GSs), which form macro polymers through disulfide bonds, contributing to the dough’s strength and elasticity and bread-making quality [2]. Gliadins, which contribute to the dough’s extensibility and viscosity [3], are classified into alpha-, gamma-, delta-, and omega gliadins based on their electrophoretic mobilities in acid polyacrylamide gel electrophoresis (A-PAGE) [4]. The omega gliadins are divided into omega-5 gliadins and omega-1,2 gliadins based on their N-terminal amino acid sequences and repetitive motifs in the central region [5]. Gamma, delta, and omega gliadins are encoded by *Gli-1* loci on the short arms of group 1 homoeologous chromosomes of hexaploid wheat, which are tightly linked to *Glu-3* loci, encoding LMW-GS. Alpha gliadins are encoded by *Gli-2* loci on the short arms of group 6 homoeologous chromosomes [6].

Gliadins, which account for approximately 30% of the total protein in wheat, are important for the functional properties that contribute to the end-use quality of flour. Unfortunately, gliadins also have immunogenic potential that can induce serious human diseases, such as celiac disease caused by epitopes in alpha, gamma, and omega-1,2 gliadins [7,8] and wheat-dependent exercise-induced anaphylaxis (WDEIA) triggered by epitopes in omega-5 gliadins [9].

To understand the complexity and heterogeneity of gliadins among different wheat varieties, considerable efforts have focused on developing methods to separate and characterize gliadin variants. The most commonly used methods for gliadin analysis are A-PAGE and sodium dodecyl sulfate–polyacrylamide gel electrophoresis (SDS-PAGE) [10,11], which are simple and easy. However, the similar mobilities of gliadins in one-dimensional PAGE make it difficult to distinguish them by banding patterns. Furthermore, contamination of samples with LMW-GS leads to difficulties such as ambiguous identification [12]. Two-dimensional gel electrophoresis provides more information than A-PAGE and SDS-PAGE, but the complicated and time-consuming procedure, high cost, and skill requirements make it difficult to use in many settings [13,14,15]. Reverse-phase–high-performance liquid chromatography (RP-HPLC) is also commonly used for gliadin analysis [16,17,18,19]. The method has sufficient resolution and is fast, highly efficient, automatable, and highly reproducible. Recently, new techniques, such as various mass spectrometry (MS) methods, have been developed to characterize gliadins [14,15,19,20,21,22,23]. Matrix-assisted laser desorption/ionization time-of-flight mass spectrometry (MALDI-TOF-MS) analysis [19,23] provides molecular weight information for each protein, and the analysis time per sample is about 4–5 min, making it suitable for high-throughput analysis. However, this method requires expensive equipment and trained personnel. As an alternative, our previous study showed that RP-HPLC is a powerful tool for gliadin analysis, showing better separation and resolution than MALDI-TOF-MS on the reference hexaploid wheat Chinese Spring (CS) and its nullisomic–tetrasomic lines (aneuploid lacking one pair of chromosomes and gaining an additional pair of homologous chromosomes) for chromosomes 1 and 6, where gliadin genes are located [19].

Recently, reverse-phase–ultra-performance liquid chromatography (RP-UPLC), an improved version of the conventional RP-HPLC technique, has been widely used to separate chemical mixtures. In RP-UPLC, particle sizes less than 2 µm can be used, providing better separation than RP-HPLC, where particle size is limited to 5 µm. In addition to providing higher resolution, the smaller particles require higher pump pressures (up to 100 MPa), making this technique very efficient and fast [24]. More recently, we have established a rapid and reliable RP-UPLC method to determine wheat HMW-GS protein profiles and validated the method in a large collection of bread wheat germplasm [25].

Of the storage proteins in wheat endosperm, gliadins are generally considered to be the most polymorphic [26]. In addition, when considering the relevance of gliadins to wheat end-use quality and human health, genetic studies of gliadins are very important. Because of the complexity and heterogeneity of these proteins, resolving the genetic polymorphisms responsible for them remains challenging. In particular, the Metakovsky group in Spain has focused on elucidating the complex variations of alleles encoding gliadins for the past 30 years. According to their A-PAGE data, wheat germplasm from various regions of the world showed tremendous polymorphism of gliadins [26,27,28,29,30,31,32,33,34,35,36,37]. In standard A-PAGE, it is possible to separate about 20–25 bands corresponding to gliadin alleles encoded at six *Gli-1* and *Gli-2* loci from a single wheat kernel based on differing electrophoretic mobility. Currently, about 180 alleles have been identified at the six *Gli* loci based on electrophoretic mobility of bands in A-PAGE [26].

The aim of our study was to develop optimized RP-UPLC methods for allelic analysis of *Gli-B1* loci, which encode omega-5 gliadins and gamma gliadins on chromosome 1B, and to validate 16 *Gli-B1* alleles reported in other studies in standard wheat genotypes [26]. We demonstrated that RP-UPLC is an effective method for rapid and reliable evaluation of genetic polymorphisms of *Gli-B1* loci related to end-use quality and human health problems in hexaploid wheat.

## 2. Results

### 2.1. Optimization of Instrument Conditions for Omega-5 Gliadin Analysis

**Figure 1:**
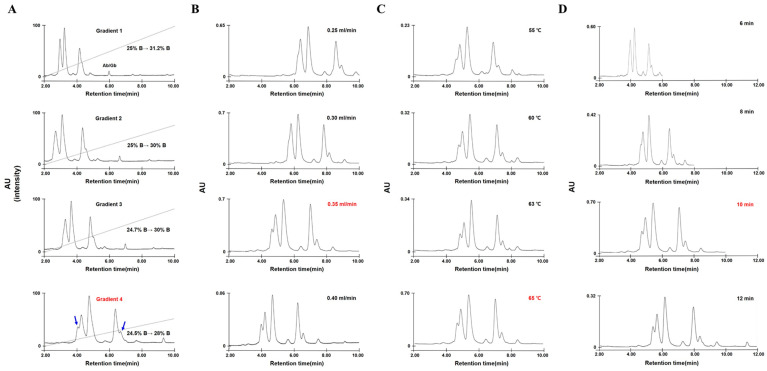
Optimization of RP-UPLC conditions for omega-5 gliadin separation. (**A**) Four solvent gradient conditions for omega-5 gliadin analysis (gradient 1: 25–31.2% solvent B (ACN), gradient 2: 25–30% solvent B, gradient 3: 24.7–30% solvent B, and gradient 4: 24.5–28% solvent B). (**B**) Separation flow rate (0.25, 0.30, 0.35, and 0.40 mL/min). (**C**) Column temperature (55 °C, 60 °C, 63 °C, and 65 °C). (**D**) Running time (6, 8, 10, and 12 min) for a linear increase in solvent B from 24.5 to 28%. Red: optimized conditions, blue arrows: reference peaks for determining optimal conditions, dashed line: linear gradient of solvent B.

Total gliadins extracted from flour of the reference wheat cultivar Chinese Spring (CS) were analyzed using our previously published RP-UPLC method [25], revealing that omega-5 gliadins, the most hydrophilic among the gliadin fractions and encoded by *Gli-B1* loci on the B1 chromosome, were eluted between 2 and 5 min. To improve their separation efficiency and peak resolution, we attempted to optimize essential parameters of RP-UPLC, such as the gradient mobile phase, flow rate, column oven temperature, and running time (Figure 1).

Out of four different gradients of the mobile phase solution, Figure 1A shows that omega-5 gliadins were optimally separated under gradient 4 (an acetonitrile (ACN) linear gradient from 24.5 to 28% for 10 min). Two shoulder peaks (blue arrows) in Figure 1A were found to be separated at an increased flow rate such as 0.35 and 0.40 mL/min (Figure 1B). The two peaks were also observed in the RP-UPLC system adjusted to 63 and 65 °C column oven temperatures (Figure 1C). Furthermore, similar results were observed when the time taken for solvent B to linearly increase from 24.5 to 28% was 10 and 12 min (Figure 1D). Finally, to obtain the best results even under low column pressure while reducing the operation time to analyze a large number of samples, we adjusted the RP-UPLC conditions, such as a flow rate of 0.35 mL/min, a column oven temperature of 65 °C, and a running time of 10 min.

**Figure 2:**
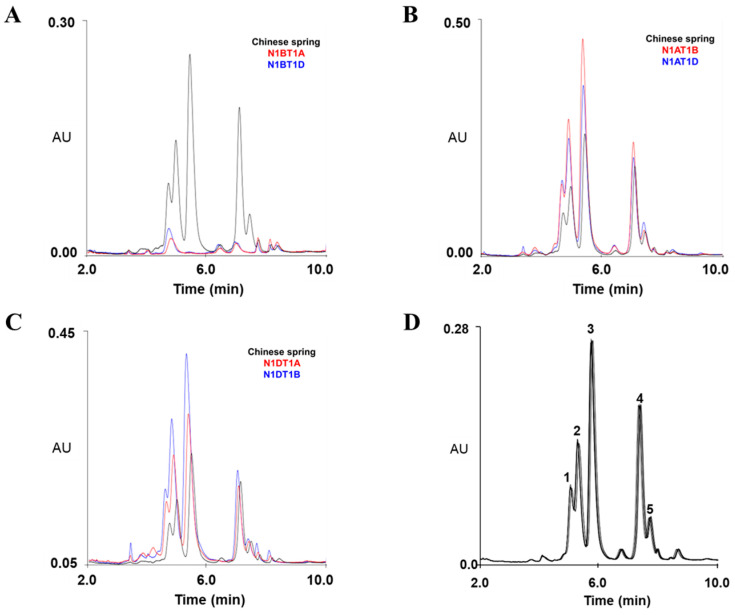
Chromatograms of omega-5 gliadins of Chinese Spring (CS) and nullisomic–tetrasomic lines of CS via RP-UPLC. (**A**–**C**) Separation of omega-5 gliadins of CS and its nullisomic–tetrasomic lines lacking A1 (N1AT1B and N1AT1D), B1 (N1BT1A and N1BT1D), and D1 (N1DT1A and N1DT1B) chromosomes using RP-UPLC. (**D**) Reproducibility of seven consecutive runs of RP-UPLC in separating five peaks of omega-5 gliadins in CS with optimized instrument conditions.

Under the optimized conditions, we analyzed the composition of omega-5 gliadins encoded by *Gli-B1* loci on the B1 chromosome from total gliadins extracted from CS and its nullisomic–tetrasomic lines (N1AT1B, N1AT1D, N1BT1A, N1BT1D, N1DT1A, and N1DT1B), which are aneuploid strains of CS. Unlike in CS and its four nullisomic–tetrasomic lines lacking the A1 (N1AT1B and N1AT1D) or D1 chromosomes (N1DT1A and N1DT1B), the five peaks of omega-5 glutelins were not observed in two lines (N1BT1A and N1BT1D) lacking the B1 chromosome, indicating that the five omega-5 gliadins detected at 5.08, 5.33, 5.79, 7.43, and 7.78 min are encoded by *Gli-B1a* loci on the B1 chromosome (Figure 2 and Table 1).

We validated the accuracy and reproducibility of the optimized method using three biological replicates and seven technical replicates to separate omega-5 gliadins from total gliadins fractionated from CS wheat flour. The average relative standard deviation (RSD) of the peak retention times for each omega-5 gliadin component in CS is listed in Table 1. The RSD percentage ranged from 0.37 to 0.55% in biological replicates and from 0.31 to 0.93% in technical replicates, indicating that our optimized RP-UPLC method can rapidly and consistently separate omega-5 gliadins.

### 2.2. Characterization of Omega-5 Gliadins in Standard Common Wheat Varieties with Different Alleles at the Gli-B1 Locus

**Figure 3:**
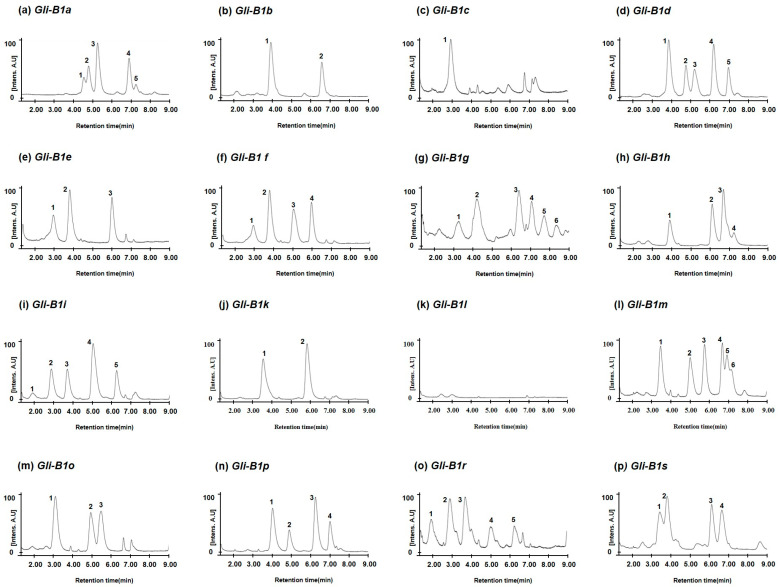
Chromatograms of omega-5 gliadins of standard wheat varieties representing each 16 *Gli-B1* allele defined using A-PAGE and SDS-PAGE. (**a**) Chinese Spring with the *Gli-B1a* allele; (**b**) Bezostaya with *Gli-B1b*; (**c**) Prinqual with *Gli-B01c*; (**d**) Neepawa with *Gli-B1d*; (**e**) Fournil with *Gli-B1e*; (**f**) Glenlea with *Gli-B1f*; (**g**) Barbilla with *Gli-B1g*; (**h**) Rudi with *Gli-B1h*; (**i**) Insignia with *Gli-B1i*; (**j**) Kremena with *Gli-B1k*; (**k**) Clement with *Gli-B1l*; (**l**) Norin 61 with *Gli-B1m*; (**m**) Aragon 03 with *Gli-B1o*; (**n**) Inia 66 with *Gli-B1p*; (**o**) Chinook with *Gli-B1r*; (**p**) Resistente with *Gli-B1s*.

To investigate whether the genetic variations in the *Gli-B1* alleles could be distinguished by the different hydrophobicities of their encoded omega-5 gliadins, we extracted total gliadins from 16 standard common wheat varieties carrying different alleles at the *Gli-B1* locus, such as Chinese Spring (*Gli-B1a* allele), Bezostaya (*Gli-B1b*), Prinqual (*Gli-B01c*), Neepawa (*Gli-B1d*) Fournil (*Gli-B1e*), Glenlea (*Gli-B1f*), Barbilla (*Gli-B1g*), Rudi (*Gli-B1h*), Insignia (*Gli-B1i*), Kremena (*Gli-B1k*), Clement (*Gli-B1l*), Norin 61 (*Gli-B1m*), Aragon 03 (*Gli-B1o*), Inia 66 (*Gli-B1p*), Chinook (*Gli-B1r*), and Resistente (*Gli-B1s*). Then, the separation of their omega gliadins via RP-UPLC (Figure 3) showed that the number and retention times of peaks corresponding to omega-5 gliadins differ in the different wheat varieties. However, the number and retention time of omega-5 gliadin peaks of Insignia (*Gli-B1i*) were five at 1.90 min, 2.91 min, 3.70 min, 5.05 min, and 6.28 min. These were very similar to those of Chinook (*Gli-B1r*) at 1.90 min, 2.85 min, 3.65 min, 5.00 min, and 6.22 min. Three omega-5 gliadin peaks in Fournil (*Gli-B1e*) at 2.95, 3.80, and 6.00 min were also detected in Glenlea (*Gli-B1f*), where four peaks were detected at 2.93, 3.80, 5.05, and 5.98 min. Four peaks in Inia 66 (*Gli-B1p*) at 4.03, 4.90, 6.25, and 7.03 min were the same as four of the five peaks in Neepawa (*Gli-B1d*) at 4.05, 4.90, 5.35, 6.28, and 7.03 min. The similarities in omega-5 gliadin composition might be related to the genetic distance between the varieties.

### 2.3. Comparative Analysis of Omega-5 Gliadin Composition in 24 Wheat Varieties with the Gli-B1f Allelxx

**Figure 4:**
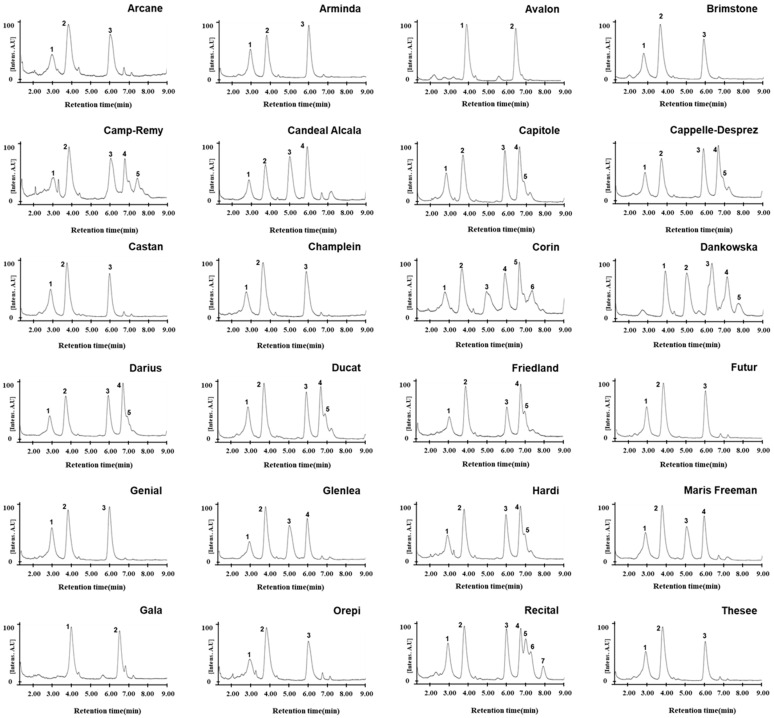
Chromatograms of omega-5 gliadins of 24 wheat varieties with the *Gli-B1f* allele. Separation of omega-5 gliadins in 24 wheat varieties via RP-UPLC. Number indicates peaks of omega-5 gliadin.

Differences in wheat varieties containing the *Gli-B1e* or *Gli-B1f* alleles were determined according to the migration patterns of gamma gliadin on A-PAGE and SDS-PAGE due to an inability to distinguish their omega-5 gliadins. To investigate whether the chromatographic patterns of omega-5 gliadins obtained using RP-UPLC can be used to distinguish the *Gli-B1* alleles, we fractionized gliadins from flour in different wheat varieties grown under the same environmental conditions and then compared omega-5 gliadin peaks in 24 wheat varieties classified as having the *Gli-B1f* allele according to A-PAGE and SDS-PAGE (Figure 4). The wheat varieties were separated into eight subgroups according to the number and retention times of their omega-5 gliadin peaks (Table 2). We observed that four omega-5 gliadin peaks at 2.93, 3.80, 5.05, and 5.98 min from a standard common wheat variety, Glenlea, containing the *Gli-Bf* allele, were also present in Candeal Alcala and Maris Freeman (Group 7). Three omega-5 gliadin peaks at 2.95, 3.80, and 6.00 min were observed in nine varieties (Group 5), including Arcane, Arminda, Brimstone, Castan, Champlein, Futur, Genial, Orepi, and Thesee, whose chromatographic patterns were similar to that in Fournil, a standard common wheat variety containing the *Gli-Be* allele. Moreover, we observed five omega-5 gliadin peaks (2.90, 3.78, 5.96, 6.71, and 6.93 min) in six varieties (Group 6): Capitole, Cappelle-Desprez, Darius, Ducat, Friendland, and Hardi; five (2.95, 3.81, 6.00, 6.73, and 7.40 min) in Camp-Remy (Group 20); six (2.93, 3.79, 5.05, 6.00, 6.71, and 7.19 min) in Corin (Group 21); and seven (2.94, 3.80, 6.00, 6.72, 7.00, 7.23, and 7.91 min) in Recital (Group 23). These varieties were found to have three peaks in common that were detected in varieties of Group 7. However, two peaks in Avalon and Galad (Group 2) and five in Dankowska (Group 22) were unique and not observed in other standard common wheat varieties. The RP-UPLC analysis of omega-5 gliadin showed that wheat varieties harboring the *Gli-B1f* allele could be divided into eight subgroups and showed the presence of common omega-5 gliadins among varieties belonging to the six subgroups (Groups 5, 6, 7, 20, 21, and 23) but not two subgroups (Groups 2 and 22).

**Table 2:** Depending on the chromatographic patterns of omega-5 gliadins, 24 wheat varieties with the *Gli-B1f* allele were subdivided into eight groups.

The Number of Peaks	Retention Time (min)	Cultivars	Group *
2	3.92, 6.48	Avalon, Galad	22
3	2.95, 3.80, 6.00	Arcane, Arminda, Brimstone, Castan, Champlein, Futur, Genial, Orepi, Thesee	5
4	2.93, 3.80, 5.05, 5.98	Glenlea, Candeal Alcala, Maris Freeman	7
5	2.90, 3.78, 5.96, 6.71, 6.93	Capitole, Cappelle-Desprez, Darius, Ducat, Friendland, Hardi	6
5	2.95, 3.81, 6.00, 6.73, 7.40	Camp-Remy	20
5	3.90, 5.02, 6.34, 7.14, 7.82	Dankowska	2
6	2.93, 3.79, 5.05, 6.00, 6.71, 7.19	Corin	21
7	2.94, 3.80, 6.00, 6.72, 7.00, 7.23, 7.91	Recital	23

*: Group is defined based on the LC peak patterns of omega-5 gliadin via RP-UPLC as described in Table 3.

**Table 3:** Classification of 73 wheat varieties through RP-UPLC analysis.

Group	Variety	*Gli-B1* Allele	Group	Variety	*Gli-B1* Allele	Group	Variety	*Gli-B1* Allele
1	Chinese Spring	a	5	Lutescens 62	e	12	Clement	l
2	Avalon	f		Orepi	f		Kavkaz	l
	Bezostaya	b		Thesee	f		Seri 82	l
	Carat	b	6	Capitole	f	13	Aragon 03	o
	Creneau	b		Cappelle-Desprez	f		San Rafael	o
	Gabo	b		Darius	f	14	Norin 61	m
	Gala	f		Ducat	f		Titien	m
	Mentana	k		Friedland	f	15	Marquis	b
	Partizanka	b		Hardi	f	16	Prinqual	c
	Renan	b	7	Candeal Alcala	f	17	Chopin	d
	Rivoli	b		Glenlea	f	18	Suneca	d
	Soissons	b		Maris Freeman	f	19	Apexal	e
3	Neepawa	d	8	Champtal	g	20	Camp-Remy	f
	Petrel	d		Mara	g	21	Corin	f
4	Yecora	d		Sadovo	g	22	Dankowska	f
	Yecora Rojo	d	9	Canaleja	h	23	Recital	f
5	Arcane	f		Tincurrin	h	24	Barbilla	g
	Arminda	f	10	Chinook	r	25	Krasnodonka	h
	Brimstone	f		Ghurka	i	26	Rudi	h
	Castan	f		Halberd	i	27	Aradi	o
	Champlein	f		Insignia	i	28	Levent	o
	Fournil	e	11	Kremena	k	29	Montjuich	o
	Futur	f		Magnif 27	k	30	Inia66	p
	Genial	f		Pane 247	k	31	Resistente	s
				Rempart	m			

### 2.4. Classification of 73 Wheat Varieties Based on Omega-5 Gliadin Composition via RP-UPLC

Comparative analysis of the LC chromatograms of omega gliadins in 24 wheat varieties classified as having the *Gli-B1f* allele based on gliadin band patterns from A-PAGE and SDS-PAGE revealed that the varieties could be classified into eight groups (Figure 4 and Table 2). This indicates that assessing the omega gliadin composition of wheat varieties using traditional methods is limited but could be accomplished via RP-UPLC. Therefore, we tried to analyze the omega gliadin composition using RP-UPLC in 73 common wheat varieties from 18 countries with known allelic variations of the *Gli-B1* loci (Table S1), including one variety with the *Gli-B1a* allele (Chinese Spring), nine with *Gli-B1b* (Bezostaya, Carat, Creneau, Gabo, Marquis, Partizanka, Renan, Rivoli, and Soissons), one with *Gli-B1c* (Prinqual), six with *Gli-B1d* (Chopin, Neepawa, Petrel, Suneca, Yecora, and Yecora Rojo), three with *Gli-B1e* (Apexal, Fournil, and Lutescens 62), 24 with *Gli-B1f* (Arcane, Arminda, Avalon, Brimstone, Camp-Remy, Candeal Alcala, Capitole, Cappelle-Desprez, Castan, Champlein, Corin, Dankowska, Darius, Ducat, Friedland, Futur, Gala, Genial, Glenlea, Hardi, Maris Freeman, Orepi, Recital, and Thesee), four with *Gli-B1g* (Barbilla, Champtal, Mara, and Sadovo), four with *Gli-B1h* (Canaleja, Krasnodonka, Rudi, and Tincurrin), three with *Gli-B1i* (Ghurka, Halberd, and Insignia), four with *Gli-B1k* (Kremena, Magnif 27, Mentana, and Pane 247), three with *Gli-B1l* (Clement, Kavkaz, and Seri 82), three with *Gli-B1m* (Norin 61, Rempart, and Titien), five with *Gli-B1o* (Aradi, Aragon 03, Levent, Montjuich, and San Rafael), one with *Gli-B1p* (Inia66), one with *Gli-B1r* (Chinook), and one with *Gli-B1s* (Resistente).

**Figure 5:**
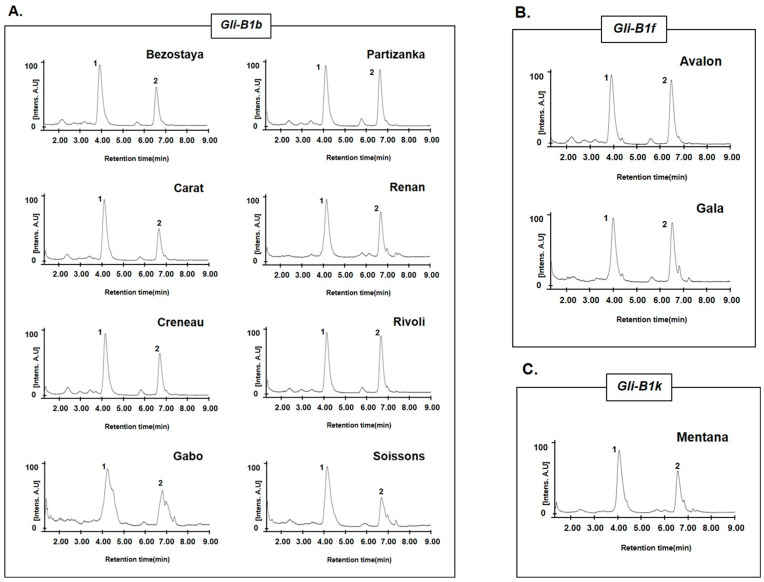
RP-UPLC analysis in wheat varieties with different *Gli-B1* alleles but similar chromatographic patterns of mega-5 gliadin. (**A**–**C**) Chromatograms of omega-5 gliadins in (**A**) eight wheat varieties with the *Gli-B1b* allele (Bezostaya, Carat, Creneau, Gabo, Partizanka, Renan, Rivoli, and Soissons), (**B**) two with the *Gli-B1f* allele (Avalon and Gala), and (**C**) one with the *Gli-B1k* allele (Mentana). Number indicates peaks of omega-5 gliadin.

**Figure 6:**
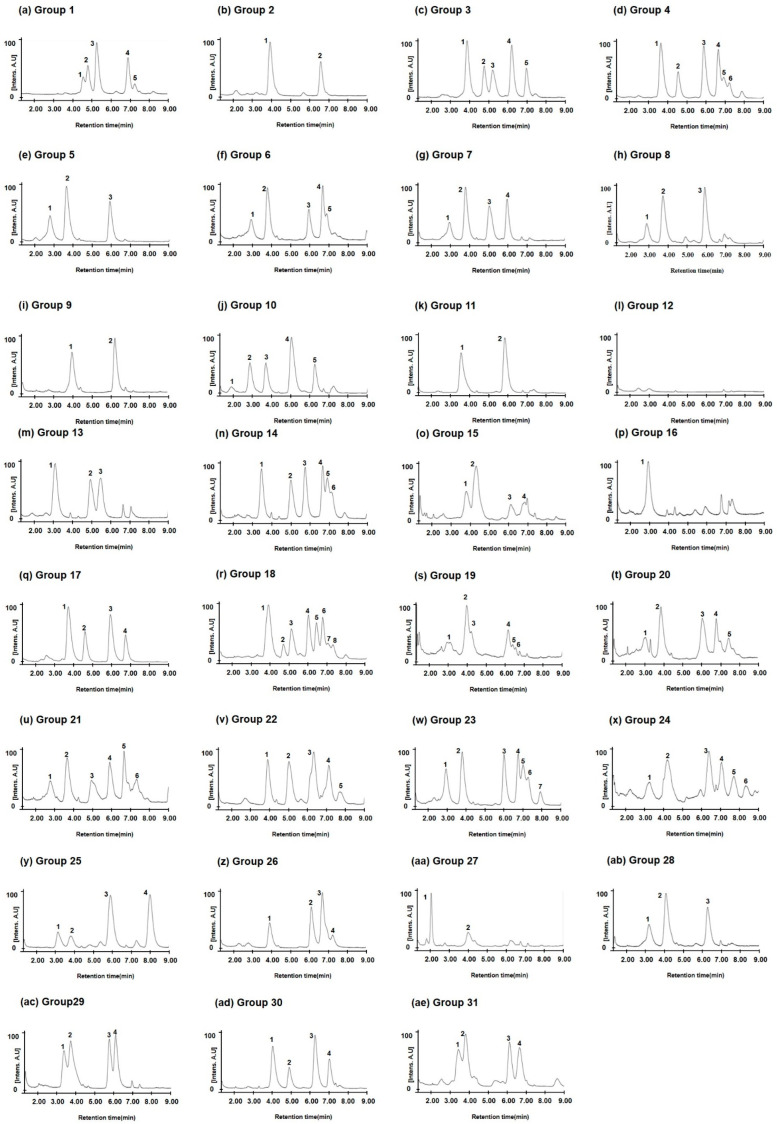
Chromatograms of omega-5 gliadin in representative wheat varieties of 31 groups classified via RP-UPLC analysis. (**a**) Chinese Spring; (**b**) Bezostaya; (**c**) Neepawa; (**d**) Yecora Rojo; (**e**) Brimstone; (**f**) Friedland; (**g**) Glenlea; (**h**) Sadovo; (**i**) Canaleja; (**j**) Insignia; (**k**) Kremena; (**l**) Clement; (**m**) Aragon 03; (**n**) Norin 61; (**o**) Marquis; (**p**) Prinqual; (**q**) Chopin; (**r**) Suneca; (**s**) Apexal; (**t**) Camp-Remy; (**u**) Corin; (**v**) Dankowska; (**w**) Recital; (**x**) Barbilla; (**y**) Krasnodonka; (**z**) Rudi; (**aa**) Aradi; (**ab**) Levent; (**ac**) Montjuich; (**ad**) Inia 66; (**ae**) Resistente. Number indicates peaks of omega-5 gliadin.

Among the nine varieties with the *Gli-B1b* allele, eight (Bezostaya, Carat, Creneau, Gabo, Partizanka, Renan, Rivoli, and Soissons; Figure 5A) had the same chromatographic peaks (4.09 and 6.65 min) for omega-5 gliadin, but one variety (Marquis) had a different pattern (3.78, 4.30, 6.12, and 6.80 min). Notably, two varieties with the *Gli-B1f* allele (Avalon and Gala; Figure 5B) and one variety (Mentana; Figure 5C) with the *Gli-B1k* allele had omega-5 gliadin chromatographic peaks similar to those of the eight varieties with the *Gli-B1b* allele. These results indicate that allelic variation at the *Gli-B1* locus could not be distinguished by the omega-5 gliadin composition of wheat varieties. To determine whether RP-UPLC analysis of omega-5 gliadins could distinguish the allelic variations at this locus, we compared the chromatographic peak patterns in 73 wheat varieties and classified the varieties into 31 groups (Figure 6 and Table 3).

Of the 31 groups, Chinese Spring with the *Gli-B1a* allele was in Group 1. Wheat varieties with two omega-5 gliadin peaks at 4.08 and 6.65 min were classified into Group 2 (Figure 5 and Figure 6). In varieties with the *Gli-B1d* allele, Groups 3 and 4 had five (4.05, 4.90, 5.35, 6.28, and 7.03 min) and six peaks (3.70, 4.58, 5.90, 6.65, 6.92, and 7.20 min) for omega-5 gliadin, respectively. As shown in Figure 4, Table 2, and Table 3, varieties with the *Gli-B1f* allele were classified into Groups 2, 5, 6, 7, 20, 21, 22, and 23 based on the chromatographic peak patterns. Moreover, three varieties (Champtal, Mara, and Sadovo) with the *Gli-B1g* allele, which had three peaks for omega-5 gliadin at 2.90, 3.75, and 5.92 min, were classified to Group 8, and two varieties (Canaleja and Tincurrin) with the *Gli-B1h* allele, which had two peaks at 3.92 and 6.15 min, were classified to Group 9. Group 10 had five omega-5 gliadin peaks at 1.92, 2.88, 3.72, 5.05, and 6.27 min in three varieties (Ghurka, Halberd, and Insignia) with the *Gli-B1i* allele and one variety (Chinook) with the *Gli-B1r* allele, and Group 11 had two peaks at 3.58 and 5.83 min in three varieties (Kremena, Magnif 27, and Pane 247) with the *Gli-B1k* allele and one variety (Rempart) with the *Gli-B1m* allele. Three varieties (Clement, Kavkaz, and Seri 82) containing *Gli-B1l*, from which no omega-5 gliadin peak was observed, were classified as Group 12. Two varieties (Aragon 03 and San Rafael) with the *Gli-B1o* allele and two varieties (Norin 61 and Titien) with the *Gli-B1m* allele were classified into Groups 13 and 14, respectively. The other varieties each belonged to a unique group with a unique chromatographic peak pattern as shown in Figure 6 and Table 3.

## 3. Discussion

Omega-5 gliadin, a component of gluten essential for bread properties, is a major allergen for individuals with wheat-dependent exercise-induced anaphylaxis (WDEIA), a rare but severe allergy exclusively occurring when wheat ingestion is accompanied by intensive exercise. The identification of epitopes in omega-5 gliadin such as QQI(F)PQQQ and QQSPQ(E)QQ and the relieved immune reactivity in WDEIA patients to omega-5 gliadin-deficient wheat compared to wild-type wheat has led to interest in the omega-5 gliadin composition of diverse wheat varieties [38,39].

The complex and polymorphic characteristics of genes encoding gliadins in allohexaploid wheat have posed many challenges to research. However, the availability of nullisomic–tetrasomic lines has clarified the genetic and chromosomal organization of omega-5 gliadin genes in wheat, revealing the presence of gliadin-coding loci (*Gli-B1*) located distally on the short arms of chromosome 1B. The mobility of gliadins on A-PAGE and the identification of gliadin genes on different chromosomes using cDNA clones and hybridization techniques revealed that *Gli-B1* loci contain several genes encoding gamma gliadins and omega-5 gliadins, which are inherited together as one trait. Metakovsky et al. [26] classified allelic types at the *Gli-B1* locus in different common wheat varieties by prioritizing the electrophoretic mobility of gamma gliadin, which showed a more pronounced difference than omega-5 gliadin on A-PAGE. This indicates that the allelic classification may not adequately describe the composition of omega-5 gliadins in different wheat cultivars.

In our previous study, we also analyzed gliadins extracted from flour of CS and its nullisomic–tetrasomic lines (N1AT1B, N1AT1D, N1BT1A, N1BT1D, N1DT1A, and N1DT1B) by using SDS-PAGE, A-PAGE, and RP-HPLC. SDS-PAGE and A-PAGE analyses indicated that genes encoding two omega-5 gliadins with different molecular weights but identical charges at acidic pH could be located at the *Gli-B1* locus. However, RP-HPLC analysis showed that at least three different omega-5 gliadins are present in CS [40], indicating that the separation of omega-5 gliadins based on hydrophobicity is more efficient than that based on molecular weight and charge. Indeed, recent studies on gliadin analysis methods have reported that the detection and quantification of gliadin proteins from wheat flour using RP-HPLC are more rapid, efficient, and accurate than with the previous methods, and RP-HPLC could fractionate gliadins with accuracy and repeatability in the following order, omega-5 gliadins, omega-1/2 gliadins, alpha/beta gliadins, and gamma gliadins, depending on hydrophobic features [41,42,43], suggesting that an RP-HPLC approach is highly useful to identify gliadin alleles in various wheat varieties. In this study, the optimized RP-UPLC analysis of gliadin extracts of CS and its nullisomic–tetrasomic lines revealed five peaks corresponding to omega-5 gliadins, which are encoded by genes located on chromosome 1B. The average RSD of each peak retention time ranged from 0.31 to 0.93% in biological and technical replicates (Table 1 and Figure 2), confirming the method’s reproducibility. These results indicate that our RP-UPLC approach is highly sensitive and efficient for omega-5 gliadin separation and useful in studying the genotypes in wheat varieties.

The analysis of omega-5 gliadins in 16 standard common wheat varieties with different alleles at the *Gli-B1* locus (Figure 3) showed that the allelic types could be discriminated according to the number and retention times of peaks corresponding to omega-5 gliadins, although similar peak patterns were observed between Insignia (*Gli-B1i*) and Chinook (*Gli-B1r*), between Fournil (*Gli-B1e*) and Glenlea (*Gli-B1f*), and between Inia 66 (*Gli-B1p*) and Neepawa (*Gli-B1d*). However, according to the LC peak patterns of omega-5 gliadins, 24 wheat varieties with the *Gli-B1f* allele were classified into eight groups (Figure 4 and Table 2). The chromatograms of omega-5 gliadins in eight wheat varieties containing the *Gli-B1b* allele were similar to those of wheat varieties with the *Gli-B1f* (Avalon and Gala) and *Gli-B1k* (Mentana) alleles (Figure 5). These results indicate that A-PAGE- and SDS-PAGE-based classification of the alleles at *Gli-B1* loci is limited by an inability to accurately distinguish the omega-5 gliadin composition. We analyzed LC chromatograms of omega-5 gliadins in 73 wheat varieties (Figure S1), which were classified into 31 groups as shown in Figure 6 and Table 3. Five wheat varieties with the *Gli-B1d* allele could be distinguished into three different groups according to LC chromatograms of omega-5 gliadins: Group 3 (Neepawa and Petrel), Group 4 (Yecora and Yecora Rojo) and Group 18 (Suneca). Twenty-four wheat varieties with the *Gli-B1f* allele were distinguished into eight groups: Group 2 (Avalon and Gala), Group 5 (Arcane, Arminda, Brimstone, Castan, Champlein, Futur, Genial, Orepi, and Thesee), Group 6 (Capitole, Cappelle-Desprez, Darius, Ducat, Friedland, and Hardi), Group 7 (Candeal Alcala, Glenlea, and Maris Freeman), Group 20 (Camp-Remy), Group 21 (Corin), Group 22 (Dankowska), and Group 23 (Recital). These results indicate that comparing retention times and peak numbers in LC chromatograms using RP-UPLC, which accurately separates omega-5 gliadins based on polarity, could allow for a more refined classification of wheat varieties than using A-PAGE to distinguish *Gli-B1* alleles. In addition to wheat cultivars with the *Gli-B1f* allele, Group 2 contains eight wheat cultivars with the *Gli-B1d* allele and Mentana with the *Gli-B1k* allele, and Group 5 includes two cultivars (Fournil and Lutescens 62) with the *Gli-B1e* allele. The fact that LC peak patterns of omega-5 gliadins in several wheat varieties with different *Gli-B1* alleles are identical, i.e., that those in varieties with the same *Gli-B1* allele are different, suggests that gamma and omega-5 gliadin genes located at the *Gli-B1* locus may not be consistently co-inherited. In other words, *Glu-B1* alleles defined based on A-PAGE analysis could not accurately indicate the composition of omega-5 gliadin in wheat varieties, but the optimized RP-UPLC method has the potential to identify omega-5 gliadins and is ideally suited for genetic studies on wheat varieties.

## 4. Materials and Methods

### 4.1. Plant Materials

A total of 73 wheat (*Triticum aestivum* L.) varieties from 18 countries with known allelic variation of the *Gli-B1* locus revealed through A-PAGE were analyzed. Among them, one variety contained *Gli-B1a*, nine contained *Gli-B1b*, one contained *Gli-B1c*, six contained *Gli-B1d*, three contained *Gli-B1e*, 24 contained *Gli-B1f*, four contained *Gli-B1g*, four contained *Gli-B1h*, three contained *Gli-B1i*, four contained *Gli-B1k*, three contained *Gli-B1l*, three contained *Gli-B1m*, five contained *Gli-B1o*, and one variety each contained *Gli-B1p*, *Gli-B1r*, or *Gli-B1s* (Table S1). The seeds of grain samples used in the study were obtained from the National Plant Germplasm System (NPGS, https://www.ars-grin.gov/npgs/ (accessed on 5 March 2021)) in the USA and the National Agrobiodiversity Center (http://genebank.rda.go.kr/ (accessed on 10 February 2023)) in Republic of Korea. Seeds of the reference wheat cultivar Chinese Spring and its six chromosome 1 aneuploid lines (N1AT1B, N1AT1D, N1BT1A, N1BT1D, N1DT1A, and N1DT1B) used as controls for RP-UPLC analysis of the *Gli-B1* allele were provided by the National Bioresource Project-wheat (NBRP-Wheat, https://shigen.nig.ac.jp/wheat/komugi/ (accessed on 9 April 2021)) in Japan. For this study, seed samples were harvested from wheats grown at the National Institute of Agricultural Sciences, Jeonju, South Korea, in 2017 as previously reported [19]. All samples used in the experiment were crushed with a cyclone sample mill (Udy Corporation, Fort Collins, CO, USA) and were turned into flour.

### 4.2. Gliadin Extraction

Gliadin fractions were prepared according to the procedure described by Jang et al. [19] with modifications. First, 50 mg of wheat flour was dissolved in 500 μL of 0.4 M NaCl solution containing 0.067 M HKNaPO_4_, followed by centrifugation at 12,500 rpm for 10 min at room temperature (RT). After discarding the supernatant containing albumin and globulin, the pellet with gliadins was dissolved with 500 μL of 60% ethanol and shaken for 30 min at RT. After centrifugation at 12,500 rpm for 10 min at RT, the supernatant containing the gliadins was transferred to a new 1.5 mL tube. Extraction was repeated twice. Total gliadin-extracted solution (1 mL) was freeze-dried overnight and stored at –80 °C until use.

### 4.3. RP-UPLC Analysis

The fraction of omega-5 gliadins from total gliadin extracts was analyzed with RP-UPLC using an ACQUITY UPLC H-Class System (Waters Corp, Milford, MA, USA) equipped with an ACQUITY UPLC Peptide BEH C_18_ Column (Waters Corp, particle size 1.7 µm, 2.1 × 100 mm id, pore size 300 Å). The mobile phases consisted of solvent A (analytical grade water: 0.06% trifluoroacetic acid (TFA)) and solvent B (acetonitrile (ACN): 0.06% TFA). The UPLC gradient was applied at a flow rate of 0.35 mL/min as follows: Solvent B was linearly increased from 24.5% at 0 min to 28% at 10 min. The column oven temperature was set to 65 °C and the elution of omega-5 gliadins was monitored at 210 nm. To optimize the separation of omega-5 gliadins encoded by the *Gli-B1* locus, RP-UPLC conditions were adjusted as follows: linear gradients of solvent B (gradient 1, from 25 to 31.2%; gradient 2, from 25 to 30%; gradient 3, from 24.7 to 30%; and gradient 4, from 24.5 to 28%), flow rate (0.25, 0.30, 0.35, and 0.40 mL/min), oven temperature (55, 60, 63, and 65 °C), and running time (6, 8, 10, and 12 min). The freeze-dried gliadin sample was mixed in 400 μL of 20% ACN solvent with 0.06% TFA and then filtered using a PVDF syringe filter (0.22 µm, Whatman, Buckinghamshire, UK). The injection volume was 5 μL. All analyses were conducted in triplicate.

### 4.4. Statistical Analysis

To test the reproducibility of the analysis conditions, Chinese Spring was analyzed five times, and according to these data, the mean, standard deviation (*p* < 0.05), and relative standard deviation% (RSD%) were determined using Excel 2019 (Microsoft, Redmond, DC, USA).

## Figures and Tables

**Table 1:** Reproducibility of seven replicates of RP-UPLC to separate omega-5 gliadins in CS.

Gliadins	Peak No.	Retention Time (min)	RSD% ^1^	RSD% ^2^
Omega-5	1	5.082 * ± 0.024 **	0.465	0.932
	2	5.329 ± 0.029	0.551	0.831
	3	5.790 ± 0.027	0.475	0.641
	4	7.428 ± 0.031	0.418	0.365
	5	7.777 ± 0.029	0.374	0.311

* and **: average elution time of the separate gliadin peaks and *p* < 0.05%, respectively. RSD% ^1^ and RSD% ^2^ were the percentage of average relative standard deviations calculated by analyzing three biological and seven instrumental replicates, respectively.

## Data Availability

The original contributions presented in this study are included in the article/Supplementary Material. Further inquiries can be directed to the corresponding authors.

## Supplementary Materials

The following supporting information can be downloaded at: https://www.mdpi.com/article/10.3390/molecules30030609/s1. Figure S1: Analysis of ω5-gliadins and γ-gliadins in 73 wheat cultivars using RP-UPLC. Alleles of the *Gli-B1* locus are grouped based on the ω5-gliadins peaks. Gliadins encoded by the *Gli-B1* locus are indicated by numbers; Table S1: Classification of Gli-B1 alleles encoding ω5-gliadins in 73 wheat cultivars.
