# Supplementary material for: Allelic Analysis of the Gli-B1 Locus in Hexaploid Wheat Using Reverse-Phase–Ultra-Performance Liquid Chromatography
Source: Molecules. 2025 Jan 30;30(3):609. doi: 10.3390/molecules30030609 (PMC11821039; doi:10.3390/molecules30030609)
Supplement: Supplementary file 1 [file molecules-30-00609-s001.zip › Figure S1.pptx]

## Slide 1
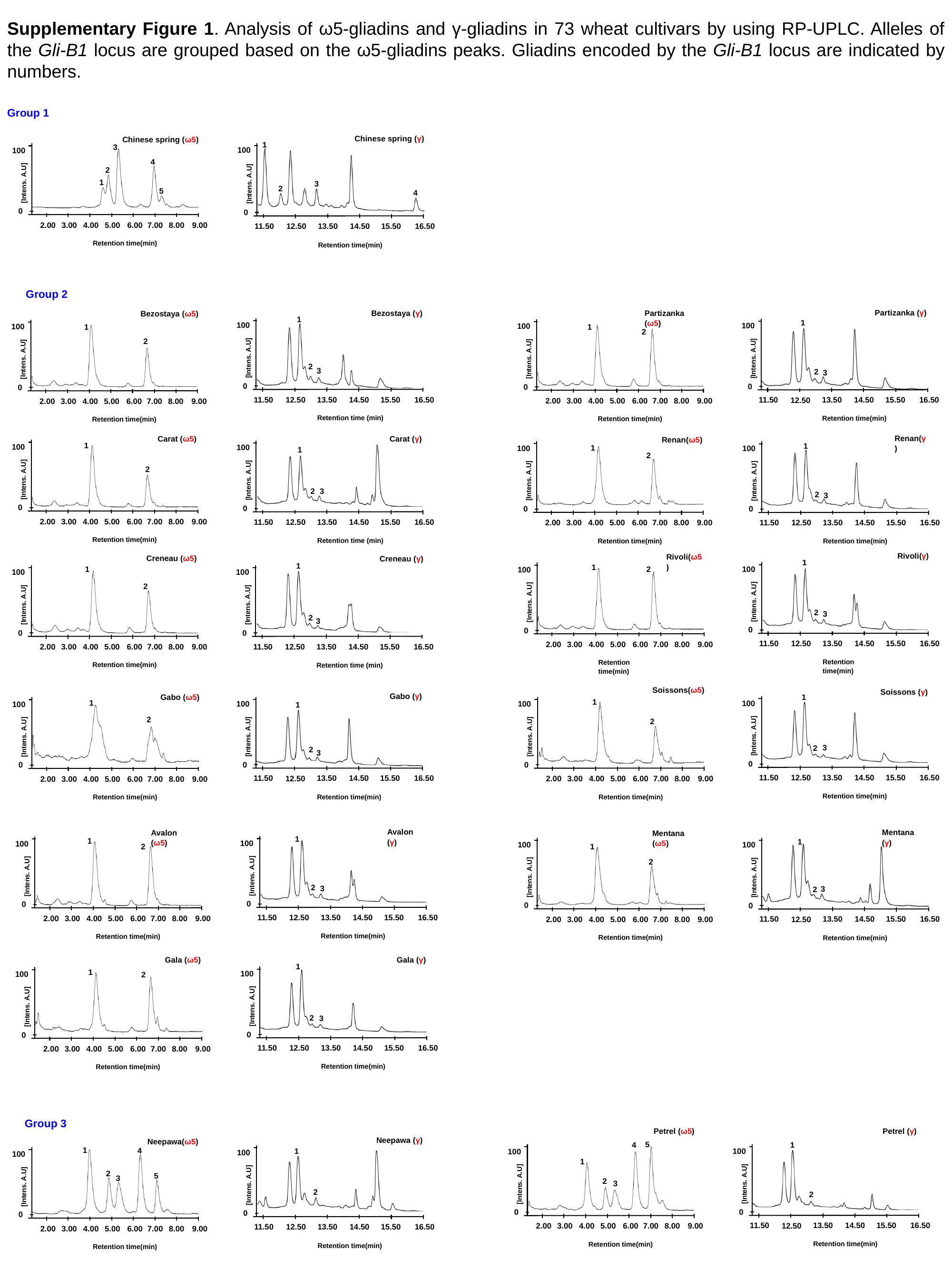

Supplementary Figure 1. Analysis of ω5-gliadins and γ-gliadins in 73 wheat cultivars by using RP-UPLC. Alleles of the Gli-B1 locus are grouped based on the ω5-gliadins peaks. Gliadins encoded by the Gli-B1 locus are indicated by numbers.
Group 1
100
0
[Intens. A.U]
11.50
13.50
14.50
15.50
16.50
Retention time(min)
Chinese spring (γ)
1
3
2
4
12.50
Chinese spring (ω5)
3
100
4
2
1
[Intens. A.U]
5
0
2.00
3.00
4.00
5.00
6.00
7.00
8.00
9.00
Retention time(min)
Group 2
100
0
[Intens. A.U]
11.50
13.50
14.50
15.50
16.50
Retention time(min)
Partizanka (γ)
1
2
12.50
3
Partizanka (ω5)
100
[Intens. A.U]
0
2.00
3.00
4.00
5.00
6.00
7.00
8.00
9.00
Retention time(min)
1
2
100
0
[Intens. A.U]
11.50
13.50
14.50
15.50
16.50
Retention time (min)
Bezostaya (γ)
1
2
3
12.50
Bezostaya (ω5)
100
[Intens. A.U]
0
2.00
3.00
4.00
5.00
6.00
7.00
8.00
9.00
Retention time(min)
1
2
100
0
[Intens. A.U]
11.50
13.50
14.50
15.50
16.50
Retention time(min)
Renan(γ)
1
2
12.50
3
100
0
[Intens. A.U]
11.50
13.50
14.50
15.50
16.50
Retention time (min)
Carat (γ)
1
2
3
12.50
Carat (ω5)
1
100
[Intens. A.U]
0
2.00
3.00
4.00
5.00
6.00
7.00
8.00
9.00
Retention time(min)
2
Renan(ω5)
100
[Intens. A.U]
0
2.00
3.00
4.00
5.00
6.00
7.00
8.00
9.00
Retention time(min)
1
2
100
0
[Intens. A.U]
11.50
13.50
14.50
15.50
16.50
Retention time(min)
Rivoli(γ)
1
2
12.50
3
Rivoli(ω5)
100
[Intens. A.U]
0
2.00
3.00
4.00
5.00
6.00
7.00
8.00
9.00
Retention time(min)
1
2
Creneau (ω5)
100
[Intens. A.U]
0
2.00
3.00
4.00
5.00
6.00
7.00
8.00
9.00
Retention time(min)
1
2
100
0
[Intens. A.U]
11.50
13.50
14.50
15.50
16.50
Retention time (min)
Creneau (γ)
1
2
3
12.50
Soissons(ω5)
100
[Intens. A.U]
0
2.00
3.00
4.00
5.00
6.00
7.00
8.00
9.00
Retention time(min)
1
2
100
0
[Intens. A.U]
11.50
13.50
14.50
15.50
16.50
Retention time(min)
Soissons (γ)
1
2
12.50
3
100
0
[Intens. A.U]
11.50
13.50
14.50
15.50
16.50
Retention time(min)
Gabo (γ)
1
2
3
12.50
Gabo (ω5)
100
[Intens. A.U]
0
2.00
3.00
4.00
5.00
6.00
7.00
8.00
9.00
Retention time(min)
1
2
100
0
[Intens. A.U]
11.50
13.50
14.50
15.50
16.50
Retention time(min)
Avalon (γ)
1
2
12.50
3
100
0
[Intens. A.U]
11.50
13.50
14.50
15.50
16.50
Retention time(min)
Mentana (γ)
1
2
12.50
3
Avalon (ω5)
1
100
[Intens. A.U]
0
2.00
3.00
4.00
5.00
6.00
7.00
8.00
9.00
Retention time(min)
2
Mentana (ω5)
100
[Intens. A.U]
0
2.00
3.00
4.00
5.00
6.00
7.00
8.00
9.00
Retention time(min)
1
2
100
0
[Intens. A.U]
11.50
13.50
14.50
15.50
16.50
Retention time(min)
Gala (γ)
1
2
12.50
3
Gala (ω5)
1
100
[Intens. A.U]
0
2.00
3.00
4.00
5.00
6.00
7.00
8.00
9.00
Retention time(min)
2
Group 3
Petrel (ω5)
5
4
100
[Intens. A.U]
0
2.00
3.00
4.00
5.00
6.00
7.00
8.00
9.00
Retention time(min)
1
2
3
100
0
[Intens. A.U]
11.50
13.50
14.50
15.50
16.50
Retention time(min)
Petrel (γ)
1
2
12.50
100
0
[Intens. A.U]
11.50
13.50
14.50
15.50
16.50
Retention time(min)
Neepawa (γ)
1
2
12.50
Neepawa(ω5)
1
4
100
[Intens. A.U]
0
2.00
3.00
4.00
5.00
6.00
7.00
8.00
9.00
Retention time(min)
2
5
3

## Slide 2
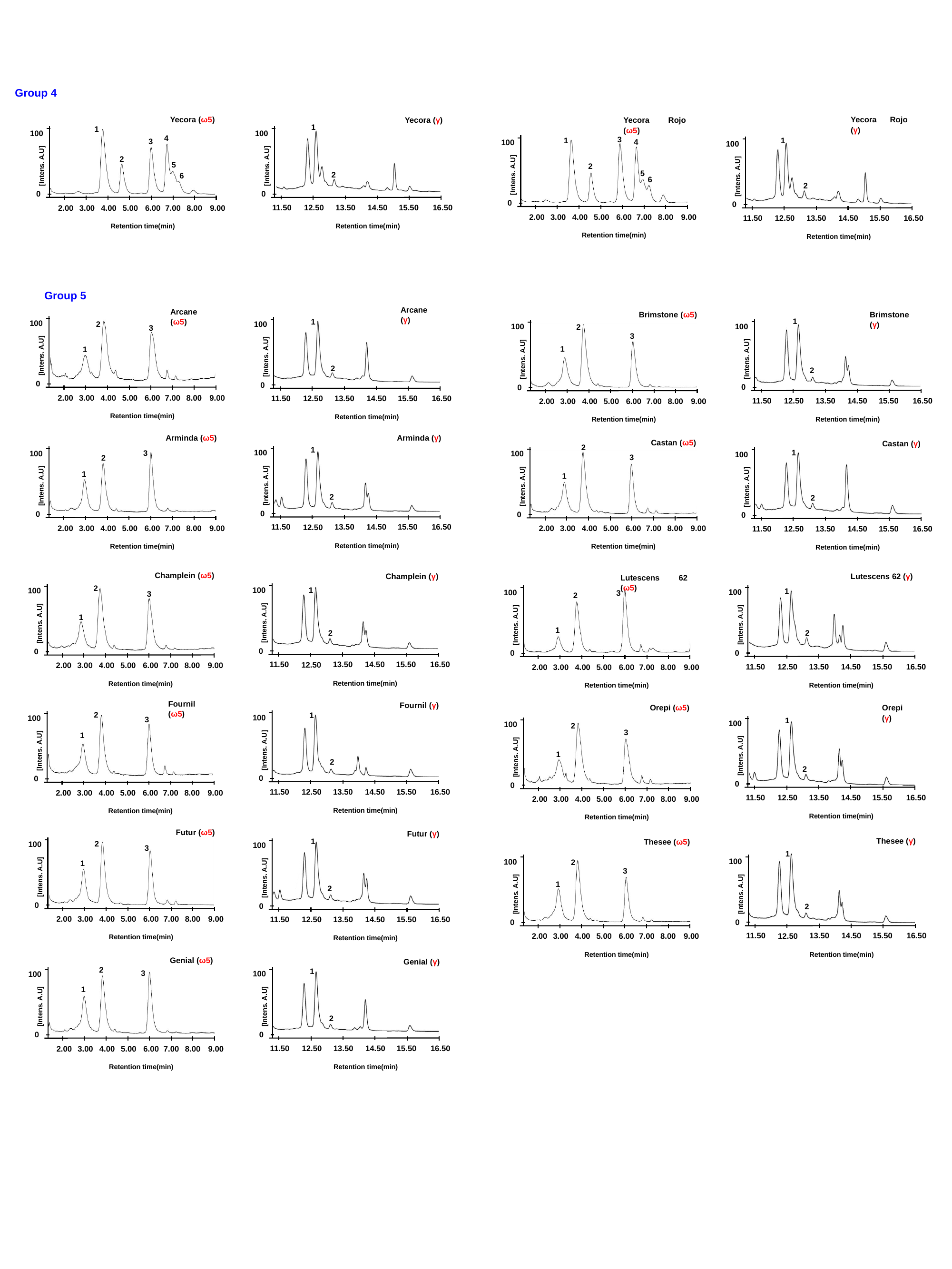

Group 4
Yecora (ω5)
1
100
[Intens. A.U]
0
2.00
3.00
4.00
5.00
6.00
7.00
8.00
9.00
Retention time(min)
4
3
2
5
6
100
0
[Intens. A.U]
11.50
13.50
14.50
15.50
16.50
Retention time(min)
Yecora Rojo (γ)
1
2
12.50
100
0
[Intens. A.U]
11.50
13.50
14.50
15.50
16.50
Retention time(min)
Yecora (γ)
1
2
12.50
Yecora Rojo (ω5)
3
1
100
[Intens. A.U]
0
2.00
3.00
4.00
5.00
6.00
7.00
8.00
9.00
Retention time(min)
4
2
5
6
Group 5
100
0
[Intens. A.U]
11.50
13.50
14.50
15.50
16.50
Retention time(min)
Arcane (γ)
1
2
12.50
Arcane (ω5)
2
3
1
100
[Intens. A.U]
0
2.00
3.00
4.00
5.00
6.00
7.00
8.00
9.00
Retention time(min)
100
0
[Intens. A.U]
11.50
13.50
14.50
15.50
16.50
Retention time(min)
Brimstone (γ)
1
2
12.50
Brimstone (ω5)
100
[Intens. A.U]
0
2.00
3.00
4.00
5.00
6.00
7.00
8.00
9.00
Retention time(min)
2
3
1
100
0
[Intens. A.U]
11.50
13.50
14.50
15.50
16.50
Retention time(min)
Arminda (γ)
1
2
12.50
Arminda (ω5)
100
[Intens. A.U]
0
2.00
3.00
4.00
5.00
6.00
7.00
8.00
9.00
Retention time(min)
3
2
1
Castan (ω5)
2
100
[Intens. A.U]
0
2.00
3.00
4.00
5.00
6.00
7.00
8.00
9.00
Retention time(min)
3
1
100
0
[Intens. A.U]
11.50
13.50
14.50
15.50
16.50
Retention time(min)
Castan (γ)
1
2
12.50
Champlein (ω5)
2
100
[Intens. A.U]
0
2.00
3.00
4.00
5.00
6.00
7.00
8.00
9.00
Retention time(min)
3
1
100
0
[Intens. A.U]
11.50
13.50
14.50
15.50
16.50
Retention time(min)
Champlein (γ)
1
2
12.50
100
0
[Intens. A.U]
11.50
13.50
14.50
15.50
16.50
Retention time(min)
Lutescens 62 (γ)
1
2
12.50
Lutescens 62 (ω5)
100
[Intens. A.U]
0
2.00
3.00
4.00
5.00
6.00
7.00
8.00
9.00
Retention time(min)
3
2
1
Fournil (ω5)
2
100
[Intens. A.U]
0
2.00
3.00
4.00
5.00
6.00
7.00
8.00
9.00
Retention time(min)
3
1
100
0
[Intens. A.U]
11.50
13.50
14.50
15.50
16.50
Retention time(min)
Fournil (γ)
1
2
12.50
Orepi (ω5)
100
[Intens. A.U]
0
2.00
3.00
4.00
5.00
6.00
7.00
8.00
9.00
Retention time(min)
2
3
1
100
0
[Intens. A.U]
11.50
13.50
14.50
15.50
16.50
Retention time(min)
Orepi (γ)
1
2
12.50
Futur (ω5)
2
100
[Intens. A.U]
0
2.00
3.00
4.00
5.00
6.00
7.00
8.00
9.00
Retention time(min)
3
1
100
0
[Intens. A.U]
11.50
13.50
14.50
15.50
16.50
Retention time(min)
Futur (γ)
1
2
12.50
100
0
[Intens. A.U]
11.50
13.50
14.50
15.50
16.50
Retention time(min)
Thesee (γ)
1
2
12.50
Thesee (ω5)
100
[Intens. A.U]
0
2.00
3.00
4.00
5.00
6.00
7.00
8.00
9.00
Retention time(min)
2
3
1
Genial (ω5)
2
3
100
[Intens. A.U]
0
2.00
3.00
4.00
5.00
6.00
7.00
8.00
9.00
Retention time(min)
1
100
0
[Intens. A.U]
11.50
13.50
14.50
15.50
16.50
Retention time(min)
Genial (γ)
1
2
12.50

## Slide 3
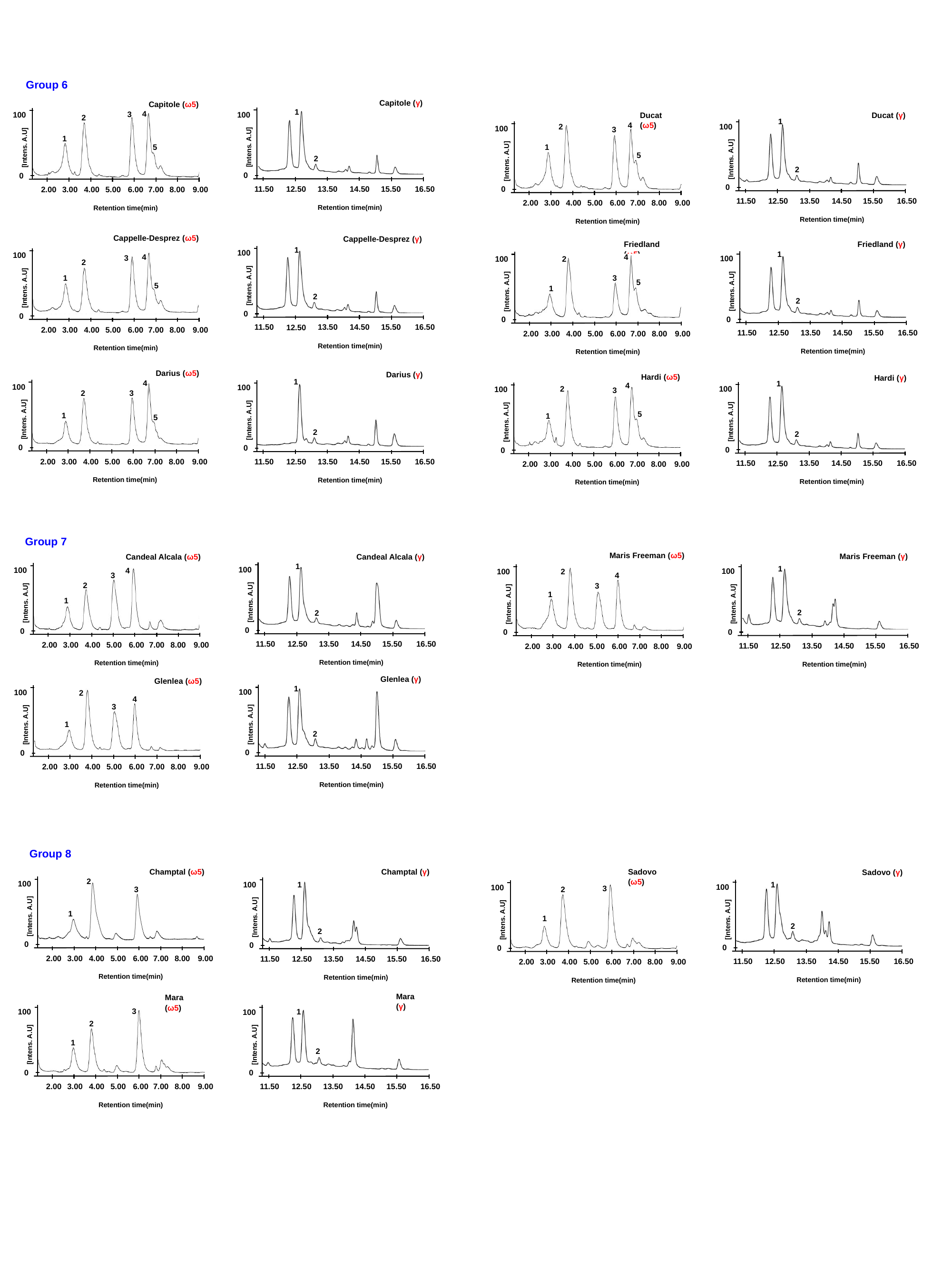

Group 6
100
0
[Intens. A.U]
11.50
13.50
14.50
15.50
16.50
Retention time(min)
Capitole (γ)
1
2
12.50
Capitole (ω5)
4
3
100
[Intens. A.U]
0
2.00
3.00
4.00
5.00
6.00
7.00
8.00
9.00
Retention time(min)
2
1
5
100
0
[Intens. A.U]
11.50
13.50
14.50
15.50
16.50
Retention time(min)
Ducat (γ)
1
2
12.50
Ducat (ω5)
4
2
100
[Intens. A.U]
0
2.00
3.00
4.00
5.00
6.00
7.00
8.00
9.00
Retention time(min)
3
1
5
Cappelle-Desprez (ω5)
100
[Intens. A.U]
0
2.00
3.00
4.00
5.00
6.00
7.00
8.00
9.00
Retention time(min)
4
3
2
1
5
100
0
[Intens. A.U]
11.50
13.50
14.50
15.50
16.50
Retention time(min)
Cappelle-Desprez (γ)
1
2
12.50
Friedland (ω5)
4
100
[Intens. A.U]
0
2.00
3.00
4.00
5.00
6.00
7.00
8.00
9.00
Retention time(min)
2
3
5
1
100
0
[Intens. A.U]
11.50
13.50
14.50
15.50
16.50
Retention time(min)
Friedland (γ)
1
2
12.50
Darius (ω5)
4
100
[Intens. A.U]
0
2.00
3.00
4.00
5.00
6.00
7.00
8.00
9.00
Retention time(min)
3
2
1
5
100
0
[Intens. A.U]
11.50
13.50
14.50
15.50
16.50
Retention time(min)
Darius (γ)
1
2
12.50
Hardi (ω5)
4
2
100
[Intens. A.U]
0
2.00
3.00
4.00
5.00
6.00
7.00
8.00
9.00
Retention time(min)
3
5
1
100
0
[Intens. A.U]
11.50
13.50
14.50
15.50
16.50
Retention time(min)
Hardi (γ)
1
2
12.50
Group 7
Maris Freeman (ω5)
100
[Intens. A.U]
0
2.00
3.00
4.00
5.00
6.00
7.00
8.00
9.00
Retention time(min)
2
4
3
1
100
0
[Intens. A.U]
11.50
13.50
14.50
15.50
16.50
Retention time(min)
Maris Freeman (γ)
1
2
12.50
Candeal Alcala (ω5)
100
[Intens. A.U]
0
2.00
3.00
4.00
5.00
6.00
7.00
8.00
9.00
Retention time(min)
4
3
2
1
100
0
[Intens. A.U]
11.50
13.50
14.50
15.50
16.50
Retention time(min)
Candeal Alcala (γ)
1
2
12.50
100
0
[Intens. A.U]
11.50
13.50
14.50
15.50
16.50
Retention time(min)
Glenlea (γ)
1
2
12.50
Glenlea (ω5)
100
[Intens. A.U]
0
2.00
3.00
4.00
5.00
6.00
7.00
8.00
9.00
Retention time(min)
2
4
3
1
Group 8
Sadovo (ω5)
100
[Intens. A.U]
0
2.00
3.00
4.00
5.00
6.00
7.00
8.00
9.00
Retention time(min)
3
2
1
100
0
[Intens. A.U]
11.50
13.50
14.50
15.50
16.50
Retention time(min)
Champtal (γ)
1
2
12.50
Champtal (ω5)
2
100
[Intens. A.U]
0
2.00
3.00
4.00
5.00
6.00
7.00
8.00
9.00
Retention time(min)
3
1
100
0
[Intens. A.U]
11.50
13.50
14.50
15.50
16.50
Retention time(min)
Sadovo (γ)
1
2
12.50
100
0
[Intens. A.U]
11.50
13.50
14.50
15.50
16.50
Retention time(min)
Mara (γ)
1
2
12.50
Mara (ω5)
3
100
[Intens. A.U]
0
2.00
3.00
4.00
5.00
6.00
7.00
8.00
9.00
Retention time(min)
2
1

## Slide 4
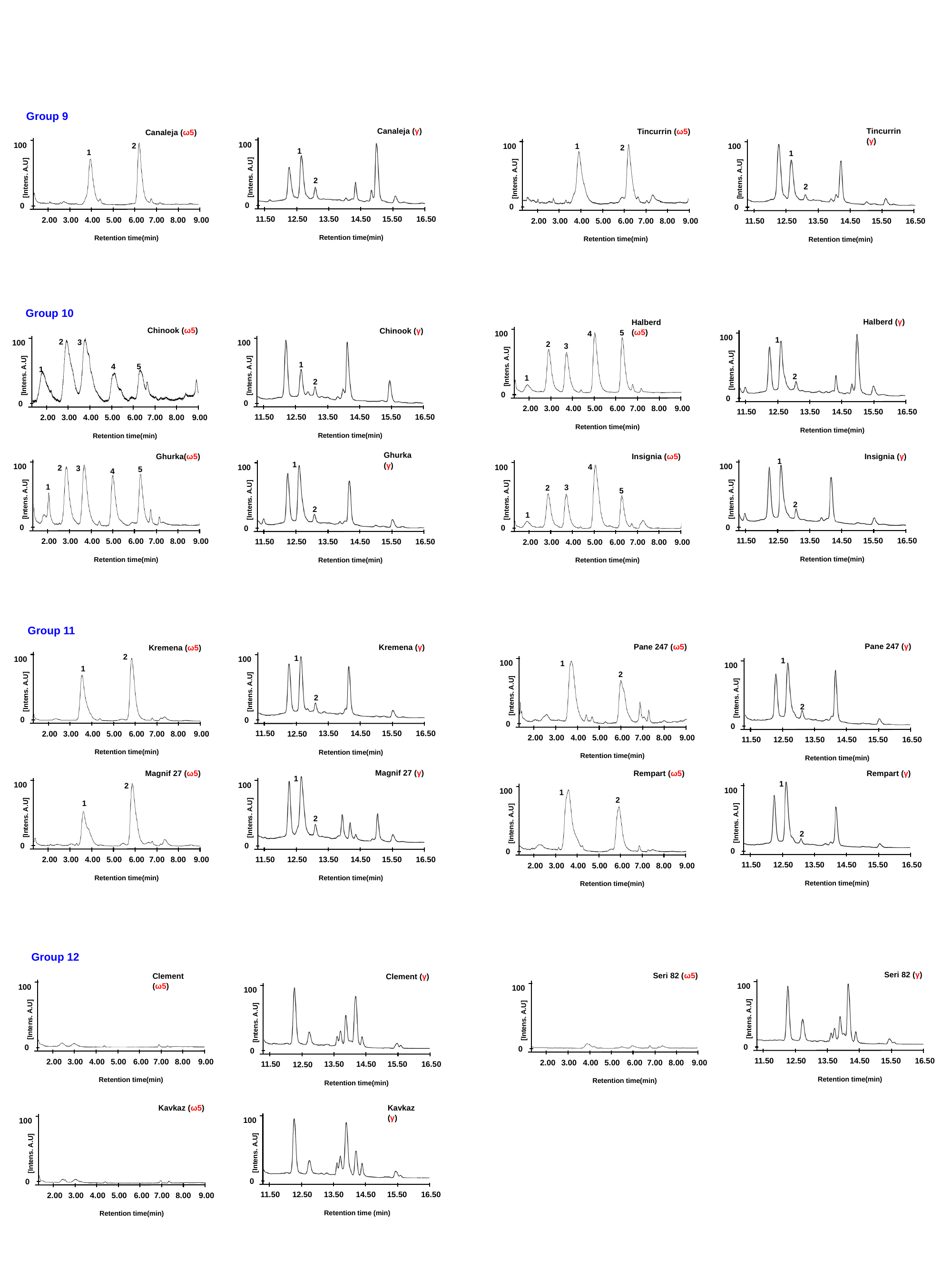

Group 9
100
0
[Intens. A.U]
11.50
13.50
14.50
15.50
16.50
Retention time(min)
Canaleja (γ)
1
2
12.50
100
0
[Intens. A.U]
11.50
13.50
14.50
15.50
16.50
Retention time(min)
Tincurrin (γ)
1
2
12.50
Tincurrin (ω5)
100
[Intens. A.U]
0
2.00
3.00
4.00
5.00
6.00
7.00
8.00
9.00
Retention time(min)
1
2
Canaleja (ω5)
100
[Intens. A.U]
0
2.00
3.00
4.00
5.00
6.00
7.00
8.00
9.00
Retention time(min)
2
1
Group 10
100
0
[Intens. A.U]
11.50
13.50
14.50
15.50
16.50
Retention time(min)
Halberd (γ)
1
2
12.50
Halberd (ω5)
5
100
[Intens. A.U]
0
2.00
3.00
4.00
5.00
6.00
7.00
8.00
9.00
Retention time(min)
4
2
3
1
Chinook (ω5)
2
3
100
[Intens. A.U]
0
2.00
3.00
4.00
5.00
6.00
7.00
8.00
9.00
Retention time(min)
4
1
5
100
0
[Intens. A.U]
11.50
13.50
14.50
15.50
16.50
Retention time(min)
Chinook (γ)
1
2
12.50
100
0
[Intens. A.U]
11.50
13.50
14.50
15.50
16.50
Retention time(min)
Ghurka (γ)
1
2
12.50
Ghurka(ω5)
100
[Intens. A.U]
0
2.00
3.00
4.00
5.00
6.00
7.00
8.00
9.00
Retention time(min)
2
3
5
4
1
Insignia (ω5)
100
[Intens. A.U]
0
2.00
3.00
4.00
5.00
6.00
7.00
8.00
9.00
Retention time(min)
4
3
2
1
5
100
0
[Intens. A.U]
11.50
13.50
14.50
15.50
16.50
Retention time(min)
Insignia (γ)
1
2
12.50
Group 11
100
0
[Intens. A.U]
11.50
13.50
14.50
15.50
16.50
Retention time(min)
Pane 247 (γ)
1
2
12.50
Pane 247 (ω5)
100
[Intens. A.U]
0
2.00
3.00
4.00
5.00
6.00
7.00
8.00
9.00
Retention time(min)
1
2
100
0
[Intens. A.U]
11.50
13.50
14.50
15.50
16.50
Retention time(min)
Kremena (γ)
1
2
12.50
Kremena (ω5)
2
100
[Intens. A.U]
0
2.00
3.00
4.00
5.00
6.00
7.00
8.00
9.00
Retention time(min)
1
100
0
[Intens. A.U]
11.50
13.50
14.50
15.50
16.50
Retention time(min)
Magnif 27 (γ)
1
2
12.50
Magnif 27 (ω5)
100
[Intens. A.U]
0
2.00
3.00
4.00
5.00
6.00
7.00
8.00
9.00
Retention time(min)
2
1
Rempart (ω5)
100
[Intens. A.U]
0
2.00
3.00
4.00
5.00
6.00
7.00
8.00
9.00
Retention time(min)
1
2
100
0
[Intens. A.U]
11.50
13.50
14.50
15.50
16.50
Retention time(min)
Rempart (γ)
1
2
12.50
Group 12
100
0
[Intens. A.U]
11.50
13.50
14.50
15.50
16.50
Retention time(min)
Seri 82 (γ)
12.50
Seri 82 (ω5)
100
[Intens. A.U]
0
2.00
3.00
4.00
5.00
6.00
7.00
8.00
9.00
Retention time(min)
Clement (ω5)
100
[Intens. A.U]
0
2.00
3.00
4.00
5.00
6.00
7.00
8.00
9.00
Retention time(min)
100
0
[Intens. A.U]
11.50
13.50
14.50
15.50
16.50
Retention time(min)
Clement (γ)
12.50
100
0
[Intens. A.U]
11.50
13.50
14.50
15.50
16.50
Retention time (min)
Kavkaz (γ)
12.50
Kavkaz (ω5)
100
[Intens. A.U]
0
2.00
3.00
4.00
5.00
6.00
7.00
8.00
9.00
Retention time(min)

## Slide 5
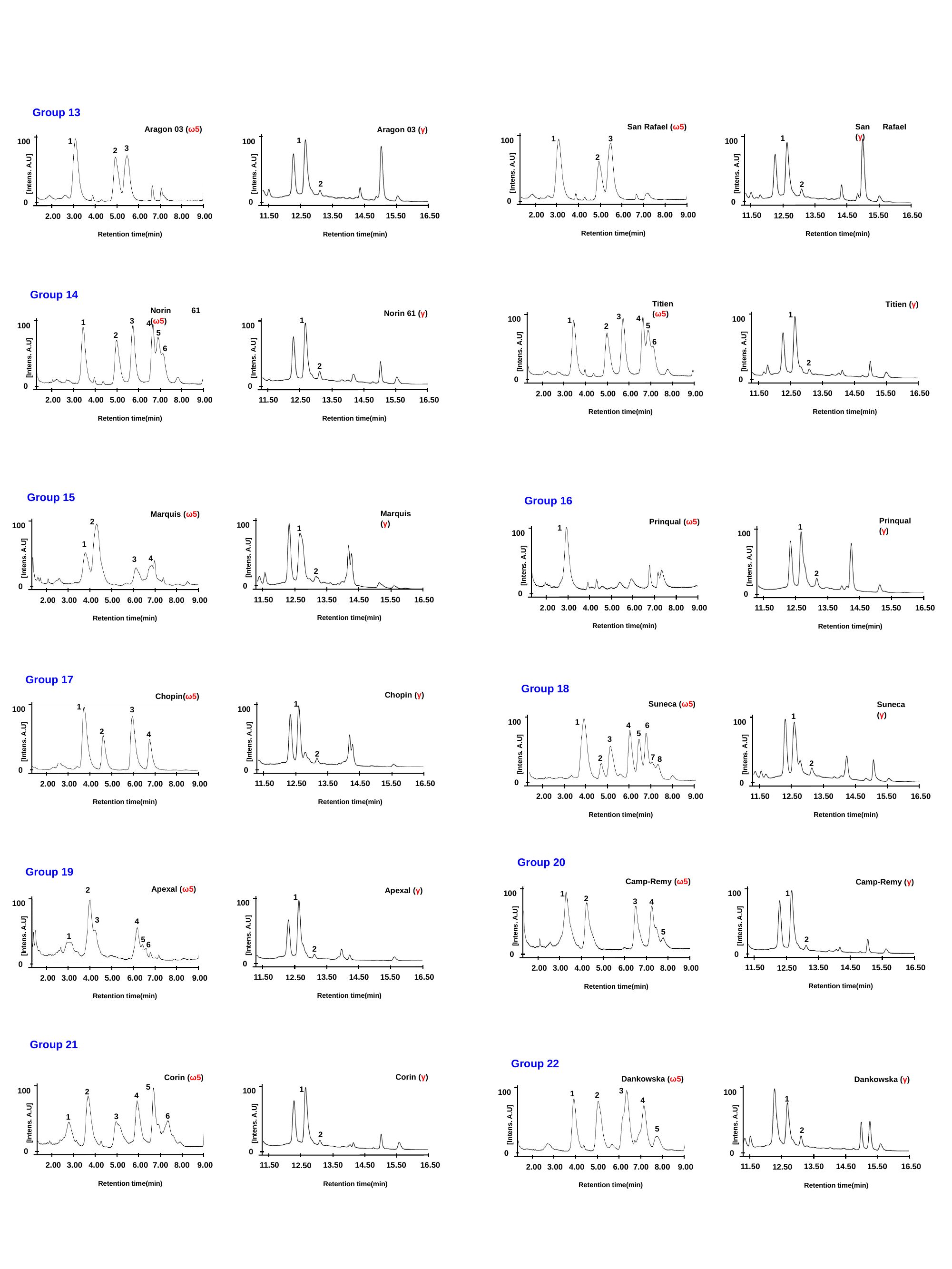

Group 13
100
0
[Intens. A.U]
11.50
13.50
14.50
15.50
16.50
Retention time(min)
San Rafael (γ)
1
2
12.50
San Rafael (ω5)
3
1
100
[Intens. A.U]
0
2.00
3.00
4.00
5.00
6.00
7.00
8.00
9.00
Retention time(min)
2
Aragon 03 (ω5)
100
[Intens. A.U]
0
2.00
3.00
4.00
5.00
6.00
7.00
8.00
9.00
Retention time(min)
1
3
2
100
0
[Intens. A.U]
11.50
13.50
14.50
15.50
16.50
Retention time(min)
Aragon 03 (γ)
1
2
12.50
Group 14
Titien (ω5)
3
4
100
[Intens. A.U]
0
2.00
3.00
4.00
5.00
6.00
7.00
8.00
9.00
Retention time(min)
1
5
2
6
100
0
[Intens. A.U]
11.50
13.50
14.50
15.50
16.50
Retention time(min)
Titien (γ)
1
2
12.50
Norin 61 (ω5)
3
1
4
100
[Intens. A.U]
0
2.00
3.00
4.00
5.00
6.00
7.00
8.00
9.00
Retention time(min)
5
2
6
100
0
[Intens. A.U]
11.50
13.50
14.50
15.50
16.50
Retention time(min)
Norin 61 (γ)
1
2
12.50
Group 15
Group 16
100
0
[Intens. A.U]
11.50
13.50
14.50
15.50
16.50
Retention time(min)
Marquis (γ)
1
2
12.50
Marquis (ω5)
2
100
[Intens. A.U]
0
2.00
3.00
4.00
5.00
6.00
7.00
8.00
9.00
Retention time(min)
1
4
3
100
0
[Intens. A.U]
11.50
13.50
14.50
15.50
16.50
Retention time(min)
Prinqual (γ)
1
2
12.50
Prinqual (ω5)
1
100
[Intens. A.U]
0
2.00
3.00
4.00
5.00
6.00
7.00
8.00
9.00
Retention time(min)
Group 17
Group 18
100
0
[Intens. A.U]
11.50
13.50
14.50
15.50
16.50
Retention time(min)
Chopin (γ)
1
12.50
2
Chopin(ω5)
1
100
[Intens. A.U]
0
2.00
3.00
4.00
5.00
6.00
7.00
8.00
9.00
Retention time(min)
3
2
4
Suneca (ω5)
100
[Intens. A.U]
0
2.00
3.00
4.00
5.00
6.00
7.00
8.00
9.00
Retention time(min)
1
4
6
5
3
7
2
8
100
0
[Intens. A.U]
11.50
13.50
14.50
15.50
16.50
Retention time(min)
Suneca (γ)
1
2
12.50
Group 20
Group 19
Camp-Remy (ω5)
100
[Intens. A.U]
0
2.00
3.00
4.00
5.00
6.00
7.00
8.00
9.00
Retention time(min)
1
2
3
4
5
100
0
[Intens. A.U]
11.50
13.50
14.50
15.50
16.50
Retention time(min)
Camp-Remy (γ)
1
2
12.50
Apexal (ω5)
2
100
[Intens. A.U]
0
2.00
3.00
4.00
5.00
6.00
7.00
8.00
9.00
Retention time(min)
3
4
1
5
6
100
0
[Intens. A.U]
11.50
13.50
14.50
15.50
16.50
Retention time(min)
Apexal (γ)
1
2
12.50
Group 21
Group 22
100
0
[Intens. A.U]
11.50
13.50
14.50
15.50
16.50
Retention time(min)
Corin (γ)
1
2
12.50
Corin (ω5)
5
100
[Intens. A.U]
0
2.00
3.00
4.00
5.00
6.00
7.00
8.00
9.00
Retention time(min)
2
4
6
3
1
Dankowska (ω5)
3
100
[Intens. A.U]
0
2.00
3.00
4.00
5.00
6.00
7.00
8.00
9.00
Retention time(min)
1
2
4
5
100
0
[Intens. A.U]
11.50
13.50
14.50
15.50
16.50
Retention time(min)
Dankowska (γ)
1
2
12.50

## Slide 6
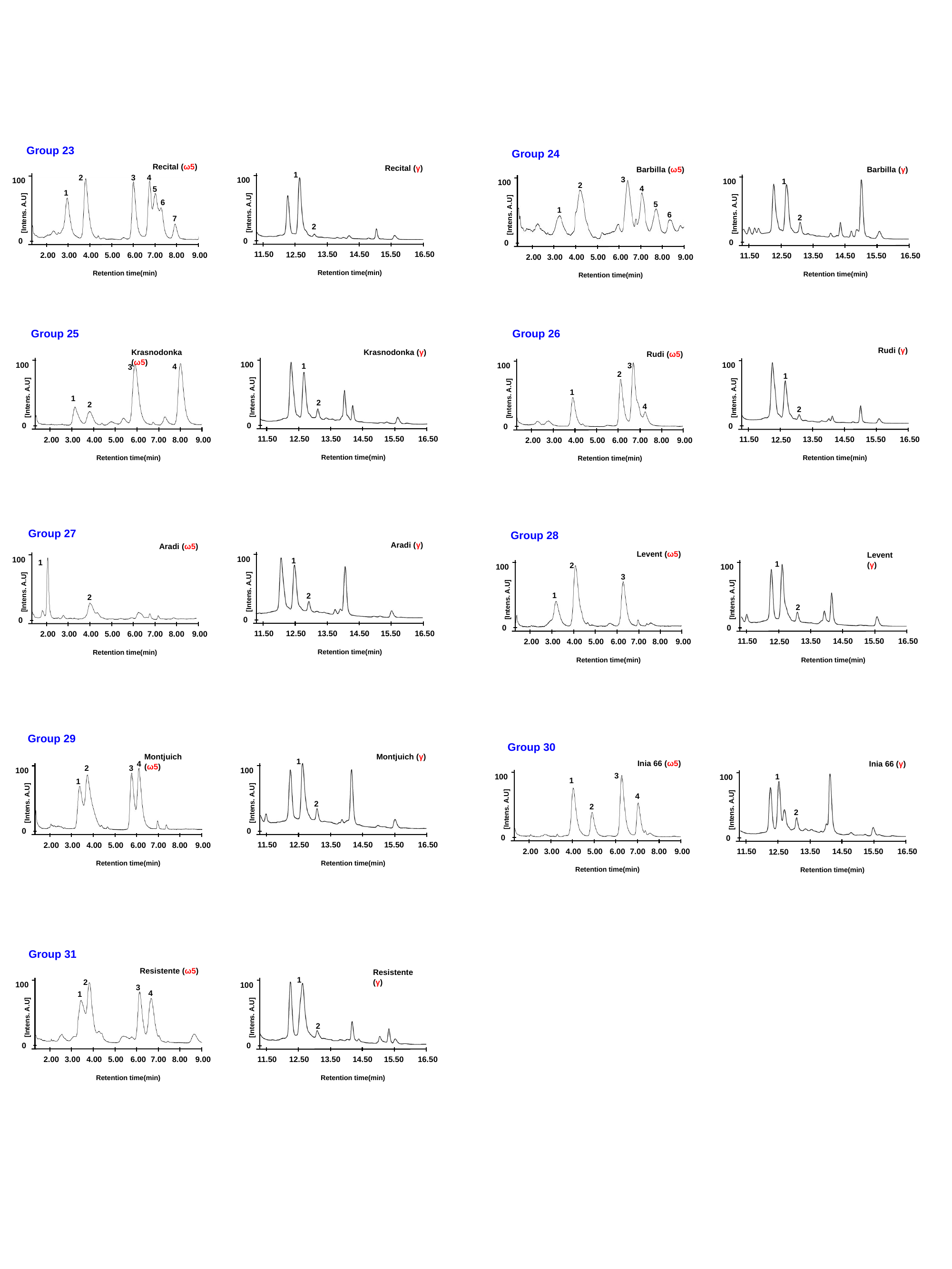

Group 23
Group 24
Recital (ω5)
2
3
4
100
[Intens. A.U]
0
2.00
3.00
4.00
5.00
6.00
7.00
8.00
9.00
Retention time(min)
5
1
6
7
100
0
[Intens. A.U]
11.50
13.50
14.50
15.50
16.50
Retention time(min)
Recital (γ)
1
2
12.50
100
0
[Intens. A.U]
11.50
13.50
14.50
15.50
16.50
Retention time(min)
Barbilla (γ)
1
2
12.50
Barbilla (ω5)
3
100
[Intens. A.U]
0
2.00
3.00
4.00
5.00
6.00
7.00
8.00
9.00
Retention time(min)
2
4
5
1
6
Group 25
Group 26
100
0
[Intens. A.U]
11.50
13.50
14.50
15.50
16.50
Retention time(min)
Rudi (γ)
1
2
12.50
Krasnodonka (ω5)
100
[Intens. A.U]
0
2.00
3.00
4.00
5.00
6.00
7.00
8.00
9.00
Retention time(min)
4
3
1
2
100
0
[Intens. A.U]
11.50
13.50
14.50
15.50
16.50
Retention time(min)
Krasnodonka (γ)
1
2
12.50
Rudi (ω5)
100
[Intens. A.U]
0
2.00
3.00
4.00
5.00
6.00
7.00
8.00
9.00
Retention time(min)
3
2
1
4
Group 27
Group 28
100
0
[Intens. A.U]
11.50
13.50
14.50
15.50
16.50
Retention time(min)
Aradi (γ)
1
2
12.50
Aradi (ω5)
100
[Intens. A.U]
0
2.00
3.00
4.00
5.00
6.00
7.00
8.00
9.00
Retention time(min)
1
2
Levent (ω5)
2
100
[Intens. A.U]
0
2.00
3.00
4.00
5.00
6.00
7.00
8.00
9.00
Retention time(min)
3
1
100
0
[Intens. A.U]
11.50
13.50
14.50
15.50
16.50
Retention time(min)
Levent (γ)
1
2
12.50
Group 29
Group 30
Montjuich (ω5)
4
2
3
100
[Intens. A.U]
0
2.00
3.00
4.00
5.00
6.00
7.00
8.00
9.00
Retention time(min)
1
100
0
[Intens. A.U]
11.50
13.50
14.50
15.50
16.50
Retention time(min)
Montjuich (γ)
1
2
12.50
Inia 66 (ω5)
3
100
[Intens. A.U]
0
2.00
3.00
4.00
5.00
6.00
7.00
8.00
9.00
Retention time(min)
1
4
2
100
0
[Intens. A.U]
11.50
13.50
14.50
15.50
16.50
Retention time(min)
Inia 66 (γ)
1
2
12.50
Group 31
Resistente (ω5)
2
100
[Intens. A.U]
0
2.00
3.00
4.00
5.00
6.00
7.00
8.00
9.00
Retention time(min)
3
4
1
100
0
[Intens. A.U]
11.50
13.50
14.50
15.50
16.50
Retention time(min)
Resistente (γ)
1
2
12.50
